# Haplotype Analysis in Carriers of *β*-Globin Gene Mutation Facilitates Genetic Counseling in *β*-Thalassemia: A Cross-Sectional Study in Kerman Province, Iran

**Published:** 2020-04

**Authors:** Nasrollah SALEH-GOHARI, Kolsoum SAEIDI, Sima ZIAADINI-DASHTKHAKI

**Affiliations:** 1. Department of Medical Genetics, Kerman University of Medical Sciences, Kerman, Iran; 2. Laboratory of Medical Genetics, Afzalipour Hospital, Kerman, Iran; 3. Neuroscience Research Center, Institute of Neuropharmacology, Kerman University of Medical Sciences, Kerman, Iran

**Keywords:** Beta-thalassemia, Beta-globin gene, Haplotype, Iran

## Abstract

**Background:**

*β*-thalassemia is characterized by reduced synthesis of the hemoglobin beta chain that results in microcytic hypochromic anemia and reduced amounts of hemoglobin A (HbA) on hemoglobin analysis. *β*-thalassemias are caused by mutations in the *β*-globin gene, inherited in an autosomal recessive manner. Determining molecular defects in couples carrying *β*-thalassemia is a prerequisite for prenatal diagnosis of the disease. In this regards, database of *β*-globin gene haplotypes facilitates mutation detection of the gene and helps genetic counselors to reach the goals of *β*-thalassemia prevention program.

**Methods:**

In this cross-sectional study, 255 couples attended genetic counseling between December 2017 and January 2019 in Afzalipour Hospital, Kerman University of Medical Scinces, Kerman, Iran as suspicious of *β-*thalassemia carriers. Furthermore, they were investigated using amplification refractory mutations system-polymerase chain reaction and restriction fragment length polymorphism methods for mutation screening and haplotype analysis of polymorphic sites in *β*-globin gene cluster, respectively.

**Results:**

We identified 20 different types of *β*-globin gene mutation in 449 *β*-thalassemia carriers. Analysis of the pattern of Hind III/Gγ, Hinf I/5′*β*, Hinc II/3′Ψ*β*, Rsa I/5′*β*, AvaII/*β* and Hind III/Aγ polymorphic sites in 257 alleles of informative families revealed 17 different haplotypes. Haplotype 1 (77.24%) showed strong linkage with the most common mutation IVSI-5 while haplotype 5 (66.67%) was associated with the second frequent mutation IVSII-1.

**Conclusion:**

To our knowledge, these *β*-globin haplotypes are reported for the first time which are different with those found in other parts of Iran. The current haplotypes pattern data makes the counseling of *β*-thalassemia carriers more straightforward and the process of mutation screening faster and more accurate.

## Introduction

Mutations in *β*-globin gene may cause *β*-thalassemia in which the syntheses of one or more globin chains are partly or completely suppressed. Depending on the nature of *β*-globin gene mutation, *β*-thalassemia is classified into two types: a) *β*-thalassemia major, also called Cooley’s anemia, is the most severe form in which affected subjects become blood transfusion-dependent and b) a milder clinical picture of the disease or thalassemia intermedia. “Patients with thalassemia intermedia present later in life with moderate anemia and do not need regular transfusion” (1). The *β*-thalassemia is inherited in autosomal recessive manner and carriers of a single *β*-globin mutation would have *β*-thalassemia minor (*β*-thalassemia trait) (2). The heterozygotes (i.e. carriers) may be slightly anemic but they are clinically asymptomatic. “Mutations of *β*-Globin gene show heterogeneity at a molecular level and usually have a geographical and ethnic distribution. More than 200 *β*-thalassemia alleles and more than 700 hemoglobin variants have been described” (3). About 52 different mutations among 20,000 homozygote patients (*β*-thalassemia major) and 3,750,000 carriers (*β*-thalassemia trait) have been identified in Iran (4). The most prevalent mutations in Iran are IVSI-5 (G>C), IVSII-I (G>A), Fr 8–9 (+G) and IVS-I-110 (G>A) (5).

Detection of *β*-globin gene mutations and prenatal diagnosis play an important role in the prevention of *β*-thalassemia. In fact, incidence of the disease in Iran has been significantly decreased since 1997 due to a national prevention program (6). The first step for premarital screening and prevention program is identification of *β*-thalassemia carriers, primarily based on the differences between the hematological indices (7). Patients with low mean corpuscular volume (MCV) and mean corpuscular hemoglobin (MCH) values, slightly reduced Hb levels (9–11.46 g/dL) and high HbA2 (HbA2 ≥3) are considered as *β*-thalassemia carriers (8).

Carrier detection is not always successful as no mutation was detected in 0.5% of *β*-thalassemia alleles neither by ARMS-PCR nor by direct sequencing (9). However, Sanger sequencing technique was used to detect unknown and rare mutations of *β*-thalassemia carriers (10). Moreover, co-inheritance of α- and *β*-thalassemia mutations which cause lower HbA2 and higher MCV and MCH levels may lead to misdiagnosis of *β*-thalassemia carriers (11). When mutation analysis fails, haplotype analysis using restriction fragment length polymorphism (RFLP) technique is useful for identifying *β*-thalassemia mutations, with an accuracy of about 90% in informative families (12). Moreover, ARMS coupled with RFLP method provides an effective technique for prenatal diagnosis of *β*-thalassemia (13).

Therefore, haplotype analysis facilitates detection of *β*-globin gene mutations and consequently the *β*-thalassemia prevention program.

Haplotype analysis of *β*-globin gene in patients with thalassemia has been reported in several populations including Korean, French-Canadians, Pakistani families and Asian-Indian race in India, Corsica Island (14–17) as well as various provinces in Iran (18–20).

*β*-thalassemia is a common genetic disorder in Kerman Province (southeast of Iran with about three million inhabitants) and the incidence rate of carriers varies in its different regions (2), therefore, using haplotype of *β*-globin gene cluster, for fast and accurate mutation detection, is of great importance. Here, for the first time, we report the association of *β*-globin gene cluster haplotypes with *β*-thalassemia mutations in Kerman Province. The obtained data helps to identify the *β*-thalassemia gene defects and increases the accuracy and quality of diagnosis.

## Materials and Methods

Red cell indices were checked in marriage registrars through public health system in Iran. In this cross-sectional study, couples with low MCV (< 82 fl) and high HbA2 (≥3), as suspicious of being *β*-thalassemia carriers or iron deficiency, was referred to our clinic for genetic counseling. These red blood cell indices were along with other low values: Hb < 12 g/dl in females and Hb<14 g/dl in males, MCH<26 pg, and mean cell hemoglobin concentration (MCHC<31 g/dl).

After ruling out iron deficiency in the selected subjects by two months iron supplementation therapy, 255 of eligible couples were further investigated for *β*-globin gene mutations between December 2017 and January 2019.

The study was approved by Ethics Committee of Afzalipour Hospital and informed consent was obtained from all participants.

**Fig. 1:**
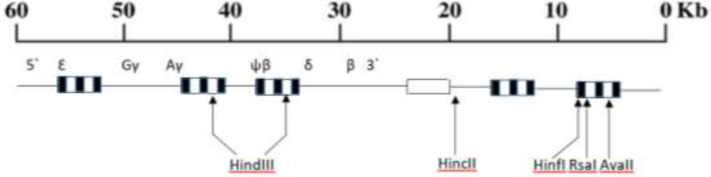
Locations of six polymorphic restriction sites within beta globin gene cluster

8 ml peripheral venous blood was collected in tubes containing Ethylene Diamine Tetra-acetic Acid (EDTA). Genomic DNA was isolated from leukocytes using salt-saturation method as previously described (21). The DNA samples were screened for the 15 most common *β*-globin gene defects by ARMS-PCR (22). Rare and unknown mutations were studied using DNA sequencing. The haplotypes study was performed using PCR amplification of *β*-globin gene cluster and RFLP techniques. DNA samples of the subjects and their parents were analyzed in order to find informative families. Primer sequences used for the amplification and detected type of the nucleotide changes identified by RFLP are shown in Table 1 and Fig. 1, respectively.

**Table 1:** Primer sequences and type of the nucleotide changes identified by RFLP. F: Forward R: Reverse

***Polymorphic site***	***Primers sequence 5′------3′***	***Nucleotide change***
Hind III/GƔ	F:AGT GCT GCA AGA AGA ACA ACT ACC R:CTC TGC ATC ATG GGC ACT GAG CTC	T>G
Hind III/AƔ	F:GAC TAG TGC TTG AAG GGG AAC AAC R:CCT CTG CTG ATT CAT TTC TTA CAC	T>G
Rsa I/*ß*	F:CCT AAT AAG TAA CTG ATG CAC AGA R:AGC TTT GGG ATA TGT AGA TGG ATC	G>T
Hinc II/Ψ*ß*	F:TAA GCA AGA TTA TTT CTG GTC TCT R:GTA CTC ATA CTT TAA GTC CTA ACT	G>C
Ava II/*ß*	F:ACT CCC AGG AGC AGG GAG GGC AGG R:TTC GTC TGT TTC CCA TTC TAA ACT	C>G
Hinf I/ *ß*	F:GGA GGT TAA AGT TTT GCT ATG R:GGG CCT ATG ATA GGG TAAT	A>T

F: Forward, R: Reverse

## Results

Out of 510 individuals participating in this research, 449 *β*-thalassemia carriers with 20 types of *β*-globin gene mutations were detected. None of them had a history of any genetic disorder except *β*-thalassemia. The remaining subjects were unknown or carriers of α-thalassemia mutations. The most prevalent mutation (58.8%) was IVSI-5 (G>C). The IVSII-I (G>A) and Hb S (A>T) were the second and the third common mutations with 8.01% and 7.57% frequencies, respectively (Table 2). All other 17 types of mutations comprise 25.62% of the total *β*-globin gene defect.

**Table 2:** Distribution of *β*-Globin Gene Mutations in Kerman Province, Iran

***Mutation***	***Number***	***%***
IVSI-5 (G> C)	264	58.8
IVSII-I (G> A)	36	8.01
Hb S (A> T)	34	7.57
Fr 8–9 (+G)	27	6.01
−88 (C>A)	15	3.34
Hb D (G>C)	12	2.68
codon 15 (G>A)	12	2.68
Codon 39 (C>T)	10	2.23
IVSI-6 (T> C)	9	2.01
Codon 44 (-C)	7	1.56
Codon 36–37 (-T)	4	0.89
619bp deletion	4	0.89
IVSI-110 (G > A)	3	0.67
IVSI-I (G > A)	2	0.44
Codon 5 (-CT)	2	0.44
*β*nt30 (T>A)	2	0.44
IVSI-25bp deletion	2	0.44
Codon 30 (G> C)	2	0.44
IVSII-745 (C->G)	1	0.23
Cap 1+ (A>C)	1	0.23
Total	449	100

**Fig. 2:**
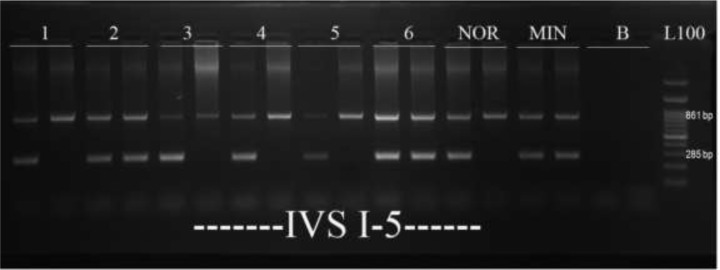
Gel electrophorese analyses of 6 samples of ARMS-PCR product related to IVS I-5 *β*-globin gene mutation. The 861bp DNA fragment is ARMS-PCR control band. The 285bp is ARMS-PCR DNA fragment produced by IVS I-5 mutation detection primers. L100; 100bp ladder, NOR; Wild type, MIN; Control heterozygote, B; blank. Samples 1, 3, 4 and 5 are wild type. Samples 2 and 6 are heterozygote.

We used RFLP technique to detect pattern of the six polymorphic sites in beta-globin gene. As an example, gel electrophoreses analysis of ARMS-PCR products of IVS I-5 *β*-globin gene mutation was shown in Fig. 2. Out of 64 possible combinations (2^6^), 17 different haplotypes (H1–H17) were found in 257 alleles (Table 3). The haplotypes of 192 uninformative alleles remained undiagnosed. Data analysis was performed by present calculation of each haplotypes and finding its relation with the *β*-globin gene mutations.

**Table 3:** Identified haplotypes of *β*-globin mutant alleles in Kerman Province

***Enzymes/ Haplotype***	***HindIII Gγ***	***HinfIβ***	***HincII β ψ***	***RsaI β***	***AvaII β***	***HindIII Aγ***
1	−	+	−	−	+	+
2	−	+	−	−	−	+
3	−	+	−	+	+	+
4	−	+	+	−	+	+
5	+	−	+	+	+	+
6	+	+	+	−	+	+
7	+	+	+	−	−	+
8	+	+	+	+	+	+
9	−	−	−	+	+	+
10	+	+	+	−	+	−
11	−	+	−	+	−	+
12	−	+	+	−	+	−
13	+	+	+	+	−	+
14	+	−	+	+	+	−
15	−	−	−	+	+	+
16	−	+	+	+	+	+
17	−	+	−	−	+	−

Haplotype 1 was the most prevalent with the frequency of 51.75% (Table 4) followed by H2 (12.6%), H3 (9.39%), H4(5.06%), H5(5.06%), and H6(4.28%). 12.4% of haplotypes were uncommon (H7 to H17). Although IVSI-5 (G-A) was present in haplotypes H1, H2, H3, H4, H6, H7, H9, H10, H13 and H17, haplotype 1 showed the strongest linkage with this mutation. The second most frequent mutation (IVSII-I) was related to 3 uncommon haplotypes; H5, H8 and H14, whereas Hb S and Fr 8–9 showed strong association with common ones; H1, H4, H5 and H1, H3, H7, respectively. The lesser frequent mutations showed linkage with one to three different haplotypes. Distribution of different identified haplotypes and their relation with 16 mutant alleles of *β*-globin gene is represented in Table 4.

**Table 4:** Distribution of different identified haplotypes in 16 mutant alleles of *β*-globin gene

***Mutation/Haplo-type***	***H1***	***H2***	***H3***	***H4***	***H5***	***H6***	***H7***	***H8***	***H9***	***H10***	***H11***	***H12***	***H13***	***H14***	***H15***	***H16***	***H17***	***Total***
IVSI-5	129 (77.24%)	17 (10.18%)	5 (3%)	1 (0.6%)	–	4 (2.39%)	4 (2.39%)	–	2 (1.2%)	2 (1.2%)	–	–	2 (1.2%)	–	–	–	1 (0.6%)	167 (100%)
HbS	1 (5%)	–	–	9 (45%)	5 (25%)	–	–	–	1 (5%)	–	–	3 (15%)	–	–	–	1 (5%)	–	20 (100%)
fr 8–9	2 (11.1%)	–	12 (66.67%)	–	–	–	4 (12.2%)	–	–	–	–	–	–	–	–	–	–	18 (100%)
IVS II-I	–	–	–	–	8 (66.67%)	–	–	2 (16.68%)	–	–	–	–	–	2 (16.68%)	–	–	–	12 (100%)
− 88(C>A)	–	–	–	–	–	7 (100%)	–	–	–	–	–	–	–	–	–	–	–	7 (100%)
Codon 39	–	6 (100%)	–	–	–	–	–	–	–	–	–	–	–	–	–	–	–	6 (100%)
IVSI-6		4 (100%)	–	–	–	–	–	–	–	–	–	–	–	–	–	–	–	4 (100%)
codon 15	–	1 (25%)	–	–	–	–	–	–	–	–	3 (75%)	–	–	–	–	–	–	4 (100%)
HbD	–	–	2 (67%)	1 (33%)	–	–	–	–	–	–	–	–	–	–	–	–	–	3 (100%)
Codon 44	–	–	2 (50%)	1 (25%)	–	–	–	1 (25%)	–	–	–	–	–	–	–	–	–	4 (100%)
IVSI-110	1 (33.3%)	–	–	1 (33.3%)	–	–	–	–	–	1 (33.3%)	–	–	–	–	–	–	–	3 (100%)
619bpdel	–	3 (100%)	–	–	–	–	–	–	–	–	–	–	–	–	–	–	–	3 (100%)
Codon 5	–	–	–	–	–	–	–	–	–	–	–	–	–	–	2 (100%)	–	–	2 (100%)
Cod 36–37	–	–	2 (100%)	–	–	–	–	–	–	–	–	–	–	–	–	–	–	2 (100%)
Codon 30	–	–	1 (100%)	–	–	–	–	–	–	–	–	–	–	–	–	–	–	1 (100%)
IVSI-25	–	–	–	–	–	–	–	1 (100%)	–	–	–	–	–	–	–	–	–	1 (100%)
Total	133 (51.75%)	31 (12.06%)	24 (9.39%)	13 (5.06%)	13 (5.06%)	11 (4.28%)	8 (3.10%)	4 (1.57%)	3 (1.16%)	3 (1.16%)	3 (1.16%)	3 (1.16%)	2 (0.77%)	2 (0.77%)	2 (0.77%)	1 (0.39%)	1 (0.39%)	257 (100%)

## Discussion

In this study, 20 different types of mutations in *β-*globin gene were identified among 449 subjects in Kerman province. The most common mutation was IVSI-5 (G>C) with a frequency of 58.8%. This finding is in agreement with reports from the south and south-east of Iran (4), including Hormozgan (23) and Sistan-and-Balochistan provinces (24). The high prevalence of the mutation can be explained either by the presence of ancestral gene defect or by gene-flow from other areas, both in combination with malaria selection (25).

The second prevalent detected mutation was IVS II-I (G>A) with a frequency of 8.3%. This mutation has also been reported to be the most common *β*-globin gene defect in Iran with the highest frequency in north of the country decreases to the south (5, 26, 27). Sickle cell trait was the third most frequent *β*-globin gene mutation (7.57%) (Table 2). The Hb S (A>T) mutation was not reported in Kerman by other surveys, whereas co-inheritance of sickle cell trait with minor *β*-thalassemia was mainly found in the south parts of the province (28).

We examined the association of 6 polymorphic restriction enzymes loci of *β*-globin gene cluster haplotypes with the gene mutations. These polymorphic sites result in 64 possible combinations, whereas we found only 17 different haplotypes (H1–H17) in 257 *β*-thalassemia mutation carriers. Ten different haplotypes were associated with the most frequent *β*-globin gene mutation (IVSI-5, G> C) in Kerman (Table 4) while this mutation was linked to 2 haplotypes in Mazandaran Province (19).

Linkage of a mutation to more than one haplotype is most consistent with the crossing-over of 5′ into the *β*-globin cluster (29). Haplotype H1 had the strongest association with IVSI-5 (G>C) mutation with the frequency of 77.24%. This helps more accurate and faster detection of this most common mutation in Kerman. Strong accompany of H1 with IVSI-5 (G>C) among 17 different haplotypes can be explained by founder effect phenomenon. The most frequent haplotype was in linkage disequilibrium with 3 other mutations with a low frequency (Table 4).

The IVS II-I (G>A) *β*-globin gene defect was associated with H5, H8, and H14 variants in which the association with haplotype H5 (66.67%) was the highest. This haplotype association also represented among the normal individuals indicating that the mutation arose on existing chromosomal background (18). Hb S has the most association with haplotype H4 by the frequency of 45%. In our knowledge, it is for the first time that the association of *β*-globin gene haplotypes and Hb S mutation is reported from Iran. Although Fr 8–9 (+G) mutation was associated with three haplotypes H1, H3, and H7, the highest connection was found with H3 (66.67%). These findings facilitate the detection of the common gene defects through haplotype studies when mutation analysis fails.

Interestingly, −88(C>A) was in linkage disequilibrium only with one haplotype (H6). As a result, detection of this haplotype confirms existence of −88(C>A) mutation with about 100% accuracy. The less common mutations; Hb D (G>C), codon 15 (G>A), codon 39 (C>T), codon 44 (-C), IVSI-6 (T>C), codon 36–37 (-T), 619bp del, IVSI-110 (G >A), codon 5 (-CT), and IVSI-25bp del were associated with 1 to 3 different haplotypes. This haplotypic heterogeneity suggest a multicentric origin of these mutations. No haplotype was linked to less common mutations; IVS I-I (Med) (G>A), −30 (T>A), IVS II-745 (C>G), and Cap +1 (A>C) in Kerman Province. We may conclude the origin of these mutations may be different from the origin of common ones.

## Conclusion

Mutations of *β*-thalassemia are frequent in Kerman province. We investigated association of *β-*globin gene cluster haplotypes with *β*-thalassemia mutations for the first time in this province. The results can facilitate the counseling and process of mutation detection in *β*-thalassemia carriers and increase the accuracy and quality of diagnosis. Similar investigation in other regions are recommended to further improve the *β*-thalassemia prevention programs and counseling.

## Ethical Consideration

All Ethical issues (such as informed consent, conflict of interest, plagiarism, misconduct, co-authorship, double submission, etc.) have been considered carefully.
